# Editorial: Innate lymphoid cell development, migration, and function

**DOI:** 10.3389/fimmu.2023.1242754

**Published:** 2023-07-03

**Authors:** Jorge Henao-Mejía, José C. Crispín, Paula Licona-Limón

**Affiliations:** ^1^ Department of Pathology and Laboratory Medicine, Perelman School of Medicine, University of Pennsylvania, Philadelphia, PA, United States; ^2^ Institute of Immunology, University of Pennsylvania, Philadelphia, PA, United States; ^3^ Division of Protective Immunity, Department of Pathology and Laboratory Medicine, Children’s Hospital of Philadelphia, Philadelphia, PA, United States; ^4^ Departamento de Inmunología y Reumatología, Instituto Nacional de Ciencias Médicas y Nutrición Salvador Zubirán, Mexico City, Mexico; ^5^ Tecnológico de Monterrey, Escuela de Medicina y Ciencias de la Salud, Monterrey, Mexico; ^6^ Departamento de Biología Celular y del Desarrollo, Instituto de Fisiología Celular, Universidad Nacional Autónoma de México, México City, Mexico

**Keywords:** innate lymphoid cell (ILC), natural killer (NK), pathology, homeostasis, heterogeneity, progenitor

Innate lymphoid cells (ILCs) represent a subset of immune cells derived from a common lymphoid progenitor. They share characteristics with classical lymphoid T cell subsets but are devoid of the antigenic presentation requirement for their activation. These cells express transcriptional regulators and effector cytokines similar to T cell subpopulations and, based on their molecular expression profile are classified into three groups: ILC1 (including NK cells), ILC2 and ILC3 (including LTi cells) (1, 2). Typically, ILCs reside in peripheral tissues and function as sentinels for pathological insults while maintaining tissue homeostasis in steady state conditions. Over the last decade, a broad spectrum of physiological conditions where ILC cells have been shown to perform critical functions has been demonstrated and growing evidence suggest they play a key, non-redundant and fundamental role in the establishment, maintenance and resolution of immune responses.

The present Research Topic includes 10 reports on key processes controlled by different types of ILCs, ranging from their development to the regulation and contribution in homeostatic and pathogenic contexts.

A broad perspective on the role of ILC subsets in different pathological contexts is discussed in two reviews by Clottu et al. and Yang et al. The first group describe functional and quantitative differences observed in ILCs in autoimmune diseases, including rheumatoid arthritis, systemic lupus erythematosus, antineutrophil cytoplasm antibody-associated vasculitis and systemic sclerosis; they also suggest the use of these cells as potential diagnostic markers and therapeutic targets. On the other hand, Yang et al. discuss cumulative evidence linking different ILC groups with the protective and pathological aspects of myocardial infarction (MI), atherosclerosis, myocardial ischemia reperfusion-injury and repair and regeneration of hearth tissue after MI. They conclude that further studies are required to dissect the contribution of different types of ILCs to be able to explore and validate their therapeutic potential in these pathologies. Lastly, a minireview by Nagashima and Iyoda expands on the possible role of ILC2s in the regulation and maintenance of renal physiology, discussing both positive and negative effects of these cells in different aspects of renal function and homeostasis maintenance.

NK cells were the first subset of ILCs described (3). They represent the cytotoxic subpopulation among ILC and accordingly, secrete perforin, granzyme and proinflammatory cytokines. A key feature of immune cells is their systemic distribution being regulated at the molecular level by different proteins coordinating migration, cytoskeletal changes, and adhesion. Myosins are some of the motor proteins involved in these processes and Cruz-Zárate et al. provide a comprehensive view of their potential role in NK cells by performing a broad analysis using public transcriptomic databases and evidence reported in other immune subsets. NK cell therapy is a promising field. Therefore, Ramos-Mejía et al. summarize the novel and functional attributes of NK cells as well as the integration of inhibitory and activation signals in different physiological contexts to exploit their therapeutic potential. Finally, by using hematopoietic specific genetic depletion of FOXO1 and FOXO3, Luu et al. explore how these transcription factors affect NK development and other non-cytotoxic ILC subsets and identify some of the molecular targets regulated by these molecules. They confirm FOXO1 and FOXO3 as transcriptional regulators controlling NK precursor commitment and development.

Growing evidence suggest a fundamental role of ILCs for tissue homeostasis. However, the current knowledge regarding the ontogeny and homing of ILC subsets to peripheral tissues, as well as specific markers able to discriminate between precursors with different origin and homing capacities *in vivo*, is still limited. In this Research Topic, Alisjahbana et al. identify CD5 as a marker of a particular subset of human ILCs present in blood and lung of humanized mice transferred with human CD34+ Hematopoietic Stem and Progenitor Cells (HSPCs) that seem to derive from a different progenitor, as suggested by its homing capacities. CD5 positive ILCs in the lung have a cytokine profile resembling ILC1s and phenotypic markers associated with ILC precursors. Given their presence in the blood, the authors propose that these cells could represent an ILC sentinel subset ready to migrate to the lung upon antigenic challenges. An important limitation to study human ILCs is the lack of efficient protocols to differentiate these cells *in vitro*. In this Research Topic, Bennstein et al. describe a method to differentiate IL-22-secreting ILC3 cells derived from CD34+ HSCPs supported by human mesenchymal stem cells (MSCs). This protocol allows for the generation of ILC3s with a molecular signature that faithfully recapitulates phenotypic hallmarks of bonafide IL-22-expressing ILC3 cells, including the expression of ROR-γt, AHR, ID2, and CD117, among others.

Finally, a detailed phenotypic characterization of ILC2s, their plasticity to respond and adapt to different microenvironments, as well as its tissue-specific heterogeneity is compiled by Olguín-Martínez et al., who emphasize the importance of using appropriate markers to identify those cells considering the pathological context and their anatomical localization. Such heterogeneity and plasticity is not restricted to ILC2s and should also be considered when studying other ILCs (4). An additional original research by the same authors uses a helminth infection mouse model to describe for the first time the phenotypic markers associated with IL-9-expressing ILC2s from the infected intestine, a tissue previously unappreciated given the difficulty to isolate and study viable ILCs. The authors then describe a protocol to generate bonafide murine ILC2s *in vitro* and identify IL-33 and the PKA pathway as key signals driving IL-9 expression and regulating functional responses in these cells.

The collection presented in this Research Topic highlights the progress made to understand and characterize the phenotype and function of ILCs in different contexts and tissues (5). It also provides new methods to study these cells *in vitro* and calls for the need of future studies to analyze their contribution and relevance in homeostatic and pathological conditions in order to explore their potential as therapeutic targets.

## Author contributions

PL-L, the guest editor, and the co-editors JH-M and JCC invited expert contributions and handled peer review. PL-L wrote the editorial with input from JH-M and JCC. All authors contributed to the article and approved the submitted version.
